# A Review of the Pathophysiology and Novel Treatments for Erectile Dysfunction

**DOI:** 10.1155/2010/730861

**Published:** 2010-07-18

**Authors:** George F. Lasker, Jason H. Maley, Philip J. Kadowitz

**Affiliations:** Department of Pharmacology, Tulane University School of Medicine, 1430 Tulane Avenue, SL83, New Orleans, LA 70112-2699, USA

## Abstract

Erectile dysfunction (ED) affects up to 50% of men between the ages of 40 and 70. Treatment with PDE-5 inhibitors is effective in the majority of men with ED. However, PDE-5 inhibitors are not effective when levels of nitric oxide (NO), the principle mediator of erection, are low. The pharmacologic actions of three new potential treatments for ED are discussed in this paper: (1) sGC stimulators/activators, (2) Rho-kinase inhibitors, and (3) sodium nitrite.

## 1. Introduction

The process of achieving penile erection involves the integration of psychological, neurological, and vascular processes, which combine to initiate a physiologic response within the penile vasculature. Endothelial mediated dilation of arteriolar smooth muscle results in increased blood flow into the sinusoids of the corpora cavernosum and subsequent filling while simultaneously relaxing to increase compliance. This filling obstructs venous outflow from the penis by compression of the veins against the tunica albuginea, resulting in penile erection.

Erectile dysfunction is defined as a difficulty in initiating or maintaining penile erection adequate for sexual relations. One of the largest current studies of ED, the Massachusetts Male Aging Study, found that ED may be present in up to half of the male population between 40 and 70 years old [1]. This condition has been estimated to affect 150 million individuals worldwide [2] and data from the ENIGMA study in 2004 suggested that the condition is prevalent in approximately 17% of all European men [3]. ED may present with comorbidities of hypertension, diabetes mellitus, obesity, and atherosclerosis [4–6]. Alcoholism, illicit drug use, and pharmacologic agents such as *β*-blockers, diuretics, and antidepressants have also been suggested to play a role in the etiology of ED [7, 8].

## 2. Characterization of ED

Erectile dysfunction can be classified as developing from psychological, neurological, hormonal, and vascular pathologies, or combinations of these factors [8]. 

### 2.1. Psychological

Psychological factors such as stress, depression, schizophrenia, and a lack of sexual arousability lead to difficulty in achieving an erection. ED may be caused by diseases that interfere with libido, and therefore the brain's perception of arousal, such as Alzheimer's, stroke, Parkinson's, or brain trauma. Injury to the spinal cord may interrupt neural pathways to the sacral region, preventing or inhibiting the process of achieving an erection [9].

### 2.2. Hormonal

Hormones such as adrenocorticotropic hormone, oxytocin, prolactin, and androgens, especially testosterone, have been implicated in the modulation of erectile function [8]. Hypogonadism plays a significant role in erectile dysfunction as it is believed that a threshold level of testosterone is necessary for erection to occur, and as men age there is a natural decrease in testosterone production further contributing to ED [10].

### 2.3. Vascular

Peripheral arterial disease and endothelial dysfunction seen in diabetes mellitus, atherosclerosis, coronary disease, and hypertension also contribute to the development of ED [11]. It has also been hypothesized that ED is an early harbinger of cardiovascular disease [12]. Along with these causes, failure to occlude venous outflow from the sinusoids of the corpora can be a contributing factor for ED. This may develop from degeneration of the tunica albuginea, loss of myogenic venous responses, trauma, or endothelial/smooth muscle dysfunction in the corpora [13].

### 2.4. Nitric Oxide and ED

NO is thought to be the main vasoactive neurotransmitter involved in the erectile response and is released from nonadrenergic, noncholinergic (NANC) neurons as well as from the endothelium [8, 14, 15]. An erection is dependent primarily upon a neurovascular, NANC mechanism peripherally, and on the central nervous system [8, 14]. Nitric oxide synthase is the enzyme responsible for the conversion of L-arginine to NO and L-citrulline. NOS has been identified within neuronal tissue (nNOS), endothelium (eNOS), and epithelial tissue within pelvic and urogenital structures of males [16, 17]. In addition to NO released from NANC nerves, shear forces also stimulate NO production by eNOS in the endothelium. NO diffuses across smooth muscle cell membrane and activates soluble guanylate cyclase (sGC), which in turn catalyzes production of cGMP from intracellular GTP. A cGMP-dependent protein kinase is activated, membrane hyperpolarization occurs through potassium channels in the smooth muscle cell membrane and there is an increase in uptake of Ca^2+^ into stores (endoplasmic reticulum). This hyperpolarization leads to blockade of membrane Ca^2+^ channels, decreasing calcium influx and causing smooth muscle cell relaxation. This relaxation produces dilation of arteries/arterioles resulting in increased blood flow into corporal sinuses in both systolic and diastolic phases. The cavernosal sinuses expand while trapping arterial inflow. Compression of the subtunical venous plexuses between the tunica albuginea and the peripheral sinusoids reduces venous outflow from the penis. Additionally, the tunica stretches to capacity and further occludes emissary veins between the inner circular and longitudinal layers further decreasing venous outflow. The partial pressure of oxygen increases from 35 mm Hg to 90 mm Hg and the intracavernosal pressure reaches approximately 100 mm Hg which raises the penis from a flaccid nonerectile state to a fully erect state (full-erection phase) [18]. Additional pressure increase results from contraction of the ischiocavernosus muscles (rigid erection phase) [19]. When the smooth muscle is then contracted, arterial inflow is reduced to a minimum and the penis assumes a flaccid state. A cGMP-specific phosphodiesterase (type 5) breaks down the cGMP to GTP and terminates membrane hyperpolarization, attenuating the relaxation of vascular smooth muscle cells.

NO is intimately involved in many of the known etiologies and comorbidities of ED. Endothelial dysfunction is caused by a decrease in formation or increase in oxidation of NO. Due to this dysfunction, the penis is not perfused sufficiently to fill the cavernosal sinusoids and cause an erection. This lack of endothelial dependent vasodilation links ED with diabetes, CVD, and hypertension- emphasizing the vital role that loss of endothelial/NO-dependent vasodilation plays in ED [11]. Reactive oxygen species (ROS) have also been implicated in type 1 diabetic ED and studies demonstrating that blockade of ROS prevent impairment of NO-mediated vasodilation [20]. Another major process linking NO and ED is impairment of NANC nerves or nNOS mediated NO release. Studies have found that type 1 diabetic animals have dysfunctional relaxation of the corpora cavernosa in response to electrical stimulation, which indicates a NANC nerve impairment [11]. Type 2 diabetic animal models have also been found to be deficient in penile nNOS [21].

## 3. Current Therapies

The current standard of care for ED consists of lifestyle changes such as management of diet, diabetes, hypertension, and weight loss, along with pharmacotherapies. The current gold standard treatment is the use of phosphodiesterase 5 inhibitors such as sildenafil citrate. As previously mentioned, phosphodiesterase 5 (PDE-5) is responsible for the breakdown of cGMP within the corpora cavernosa and the initiation of detumescence. PDE-5 inhibitors prevent this breakdown of cGMP and subsequently promote the erectile response. An individual receiving treatment for ED must be psychologically sexually stimulated to induce a NO releasing response from NANC nerve terminals initiating the sGC cascade, resulting in an increase in cGMP levels. The action of these drugs are based upon intact NO releasing neural fibers (nNOS) and corporal endothelium (eNOS). Therefore, these drugs are not effective in vascular diseases where endothelial dysfunction is significant and NO bioavailability may be impaired [7]. Sildenafil citrate was the first oral type 5 phosphodiesterase inhibitor available for treatment of ED, and has been joined on the market more recently by vardenafil and tadalafil. Sildenafil is 40% bioavailable after oral administration and is contraindicated in patients taking nitrates for angina pectoris due to the potential for a severe drop in blood pressure [22, 23]. Flushing, headache, and visual disturbances (PDE-6 inhibition) have also been reported as side effects with use of sildenafil citrate [7].

Vardenafil and tadalafil are newer in the market than sildenafil, and offer alternatives due to slight differences in their ring structures. Vardenafil, with a bioavailability of 15% after oral administration, may benefit from these structural changes through stronger binding interaction to the PDE-5 catalytic site. Tadalafil, however, differs in that the piperazine ring on vardenafil and sildenafil is completely replaced with a hydantoin ring. Crossover trials have shown that men with ED prefer tadalafil to sildenafil by up to a 9 : 1 margin [24]. This preference is increased in men with diabetes or hypertension, and is not significantly related to whether they had previously taken sildenafil [24]. Tadalafil has an increased half life, 17.5 hours, over sildenafil and vardenafil, 4-5 hours, allowing patients a longer window of time for sexual activity after taking the medication [25]. Because of this significantly prolonged half-life of tadalafil, a daily dosage may be taken so that one is always prepared for sexual activity.

## 4. Related Disease States

Despite the successful use of PDE-5 inhibitors, there is still a significant population of patients that remain refractory to this therapy. ED in individuals with chronic disease states such as diabetes mellitus (DM) and cardiovascular disease (CVD) often remains refractory because of the reliance of these drugs on functional NO release. Therefore, the examination of these disease states may provide useful insight into the etiologies of ED. 

### 4.1. Diabetes

The Massachusetts Male Aging Study (MMAS) found that diabetic men are 3 times more likely to develop ED compared to their nondiabetic counterpart [1]. In diabetic men, peripheral vasculopathy and neuropathy are intimately involved in the development of ED. Chronic hyperglycemia may lead to micro- and macrovasculopathy, including endothelial dysfunction. Autonomic and peripheral neuropathies also develop commonly in these individuals with poor glycemic control. The risk factors for diabetic ED include glycemic control, advanced age, duration of diabetes, and diabetic complications such as retinopathy. Hyperlipidemia, hypertension, and obesity are also all independent risk factors for diabetic men [26].

### 4.2. Cardiovascular Disease

Cardiovascular diseases and erectile dysfunction are closely related because both disease states involve impaired vascular endothelial function and decrease bioavailability of NO. Therefore, a high coprevalence between ED and CVD exists. Risk factors such as hypertension, hypercholesterolemia, smoking, and diabetes mellitus are also common between the two disease states [11]. It is logical that microvascular disease associated with ED should precede macrovasculopathies and studies have found that ED is significantly associated with CVD as well as CVD mortality [27, 28]. However, ED does not improve prediction of CVD beyond the traditional risk factors included in the Framingham risk score [27]. Standard therapy of PDE-5 inhibitors is strongly contraindicated in patients who are taking nitrates, as this may lead to severe hypotension and even death, thus further excluding these patients from current therapies [29]. In men with hypertension, arterial stenosis, rather than high-blood pressure, is associated with the development of ED [8]. It has been shown in spontaneously hypertensive rats that vascular relaxation, dependent on cavernosal endothelium and nitric oxide donors, is inhibited before systemic vascular changes occur. This suggests that changes to the endothelium associated with ED may precede systemic endothelial dysfunction in hypertensive patients. Oxidative damage from superoxide anion may also be important in the association between ED and hypertension [11]. In human subjects, hypertension has been correlated with a decrease in endothelial mediated smooth muscle relaxation and it has been proposed that NO may be unable to overcome the sympathetic neural activity and other procontractile mediators that are involved in establishing/maintaining the penis in a flaccid state (e.g., ET-1, neuropeptide Y, prostanoids, norepinephrine and angiotensin II, etc.) [30]. In vivo studies in rodents to model ischemia/hypertension have been performed using an iliac artery ligation to reduce perfusion [31]. This procedure results in a decrease in both the myelinated and nonmyelinated fiber diameter of nerves innervating the penis, corporal smooth muscle depletion of myofilaments and fewer endothelial cells surrounding the vasculature [31]. Because endothelial dysfunction has been associated with a variety of detrimental vascular diseases such as atherosclerosis, hypertension, and hypercholesterolemia, it was determined at the Second Princeton Consensus Conference (2006) that ED is a telltale warning sign of silent vascular disease and that a man with ED without cardiac symptoms should be considered as an “at risk” cardiovascular patient until proven otherwise [12].

### 4.3. Drug-Induced ED

It has been reported that the side effects of certain pharmacological agents may play a role in up to 25% of newly presenting ED cases [32]. Antihypertensive drugs can produce ED as a side effect. Thiazide diuretics have been reported to produce more ED than other antihypertensive agents [33]. A clear mechanism has not yet been elucidated, though it has been suggested that diuretics interfere with smooth muscle relaxation [33]. Calcium channel antagonists and ACE inhibitors have fewer detrimental effects on sexual function than diuretics, centrally acting-agents and beta-blocking therapies [34]. The aldosterone antagonist, spironolactone, is commonly prescribed for heart failure and can be used for hypertension. It can lead to ED by what is thought to be an antiandrogenic mechanism where hydrotestosterone is completely inhibited from binding androgenic receptors due to the structural similarity of spironolactone to androgens [35]. Atenolol and propranolol are two *β*-blocking agents that have been associated with ED due to their antiadrenergic effects as well as mild psychological depression resulting in reduced libido [36]. Centrally acting antihypertensives such as clonidine can act to inhibit erectile function by depressing adrenergic output. Methyldopa has a similar side effect profile in respect to ED with greater prevalence when compared to clonidine [37–40]. Many antidepressant pharmacotherapies report ED as a common side effect [8]. Increased prolactin levels associated with the use of the H_2_-antagonist cimetidine and the phenothiazine antipsychotics, chlorpromazine and thioridazine, have also been associated with ED [41–43].

### 4.4. Priapism

Priapism is defined as an erection that lasts more than 4 hours beyond sexual stimulation or that is not related to sexual stimulation [44]. The prolonged duration of erection associated with ischemic and intermittent priapism can result in destruction of sinusoidal endothelium and necrosis of cavernosal smooth muscle cells [45]. Priapism is prevalent in patients with sickle cell disease (SCD). In SCD, free hemoglobin is reported to act as a scavenging molecule oxidizing NO and forming methemoglobin [46]. The outcome of these processes is hemolytic endothelial dysfunction in which there is abnormal activity of important vasoactive signaling molecules and mechanisms such as NO, PDE-5, adenosine, and Rho-kinase. The destruction of corporal endothelium and smooth muscle that occurs with ischemic priapism often results in ED [44]. Priapism may also result from the use of erectile function promoting agents that have a long duration of action.

## 5. Need for Alternative Therapies

**Figure 1:**
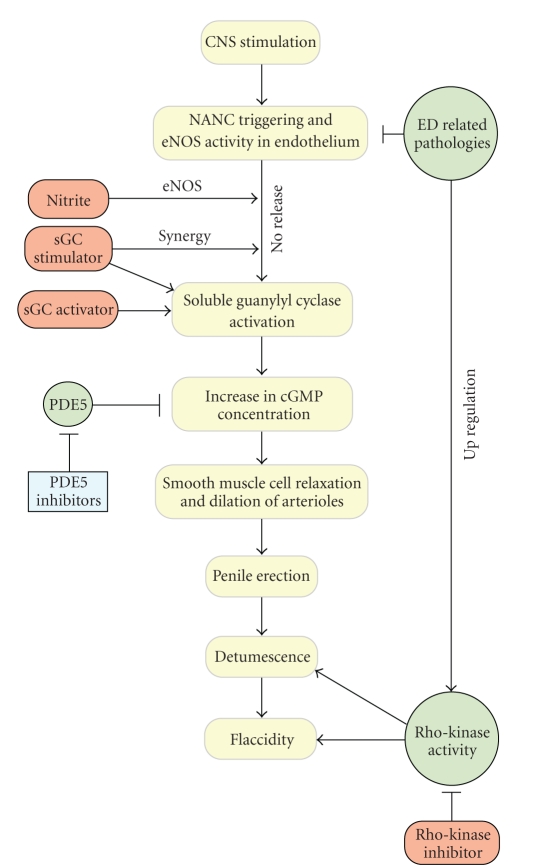
Pathway for the control of penile erection and detumescence. Stimulation of erection originates in the higher centers of the brain that result in upregulation of NANC and cholinergic activity and withdrawal of sympathetic activity in the nerves innervating the corpora cavernosa and small arteries of the penis. This increase in NANC and cholinergic activity results in upregulated NO release from the endothelium and NANC nerve terminals. The NO diffuses into the smooth muscle of the corpora cavernosa and small arteries/arterioles of the penis and binds to the reduced heme iron of soluble guanylate cyclase, activating the enzyme and increasing the formation of cGMP from GTP. cGMP-dependent protein kinase activity opens potassium channels in smooth muscle cells and increases the uptake of calcium into stores. This leads to a decrease in intracellular calcium concentration and smooth muscle cell relaxation. This increases blood flow into the corporal sinuses and the cavernosal sinuses expand trapping blood in the corpora producing a penile erection. Detumescence is initiated by release of vasoconstrictors from sympathetic terminals and endothelium. A cGMP specific phosphodiesterase (type 5) breaks down the cGMP to GTP and terminates the actions of cGMP. Three new pharmacologic targets for the treatment of erectile dysfunction (sGC stimulators/activators, Rho-kinase inhibitors and sodium nitrite) have been identified and may be effective in patients refractory to phosphodiesterase 5 inhibitor treatment. Recent experiments have shown that nitrite is capable of generating bioactive NO in the corpora cavernosa. Soluble guanylate cyclase stimulators (YC-1, A-350619, CFM-1571, and the Bayer compounds BAY 41-2272, BAY 41-8543, and BAY 63-2521) have been shown to act directly on the sGC enzyme and synergize with available NO which could be beneficial in disease states with low NO production and bioavailability. sGC activators (BAY 58-2667, BAY 60-2770 and HMR 1766/S3448) have also been shown to act on oxidized and heme-deficient sGC. Rho-kinase/RhoA activation has been shown to mediate detumescence and maintain flaccidity. Rho kinase inhibits the regulatory subunit of myosin phosphatase within smooth muscle cells and maintains contractile tone under low-cytosolic calcium concentrations. Upregulated Rho-kinase activity has been reported in ED, so Rho-kinase inhibitors (Y-27632 and SAR 407899) have potent erectile effects and offer another therapeutic target for the treatment of ED.

Pathologic conditions involving impaired NO synthesis can lead to ED that is refractory to treatments with PDE-5 inhibitors. Clinical trials for the PDE-5 inhibitor sildenafil reported that up to 40% of patients with diabetes and 50% of patients postprostatectomy did not respond to treatment [47]. In these conditions a small amount of NO is released and activation of sGC is minimal so that adequate levels of cGMP to facilitate erection are not reached [48]. The aim of this paper is to discuss 3 types of new pharmacotherapies that have surfaced in the last decade and may provide an alternative to current options for treatment of ED in the future. These emerging treatments are summarized in Figure 1 along with the pathway mediating erection.

## 6. sGC Stimulators/Activators

Nitric oxide is the key mediator of the erectile process. Under normal physiologic conditions activation of the heterodimeric heme protein soluble guanylyl cyclase (sGC) is based upon the interaction of NO with a heme iron, breaking of an Fe–His bond and a change in the conformation of sGC to its active form. It has been demonstrated that activity can occur in an NO-independent manner with protoporphyrin IX [49–51]. The need for therapies that bypass the NO stimulating step but can still activate the sGC-cGMP pathway has led to the development and use of sGC stimulators and activators.

In 1994, a team led by Feng-Nien Ko reported on a benzylindazole compound, YC-1 [3-(5′-hydroxymethyl-2′-furyl)-1-benzylindazole], that prolonged tail bleeding time of conscious mice, demonstrated direct guanylate cyclase activation in rabbit platelets, and increased cGMP levels independent of NO [52]. YC-1 has vasodilator activity and the compound synergizes with NO to increase vasodilatory responses to NO donors [53]. YC-1 was shown to have erectile activity in the rat after intracavernosal (ic) injection and also to enhance erectile responses to apomorphine and cavernous nerve stimulation when administered systemically [54]. The research with the first sGC stimulator provided a basis for the development of the next generation stimulators with improved potency and sGC specificity including CFM-1571, and the Bayer compounds BAY 41-2272, BAY 41-8543 and BAY 63-2521 [55–58].

Experiments with BAY 41-2272 and the heme-removal detergent Tween-20 showed that the sGC stimulator has no bioactivity on a heme-free sGC enzyme [56]. It was also suggested through photo-affinity labeling that the BAY compound does not directly bind the heme moiety but instead interacts with the *α*_1_-unit of sGC [56]. It was determined that BAY 41-2272 increased sGC activity 20 fold over baseline in the absence of NO and the response was potentiated further with the addition of the NO donor DEA/NO [56]. sGC stimulators can act independently of NO as well as synergize with NO, however, they require a reduced heme moiety for bioactivity [59]. The mechanism of action for these stimulators has not been completely elucidated; however, it has been proposed that NO-YC-1 synergy exists because the stimulator maintains the active conformation of sGC through stabilization of the nitrosyl-heme complex [60–62]. It was shown that oxidized derivatives of both BAY 41-2272 and BAY 41-8543 remained bioactive in experiments with rats and dogs, suggesting a role for the metabolites of these sGC stimulators and their potential residual benefit in vivo [63].

The properties of another novel sGC stimulator, A-350619, were investigated in a conscious Wistar rat model and induced penile erection after intraperitoneal injection [64]. BAY 41-2272 was administered to rabbits and initiated a small erectile response similar to sildenafil which was hypothesized to be weak due to lack of sexual stimulation [65, 66]. The erectile responses to BAY 41-2272 were significantly enhanced with concurrent administration of the NO donor sodium nitroprusside (SNP), demonstrating a synergy between the compound and NO [67]. A comparative study of sildenafil, an NO releasing sildenafil agent (NCX-911), and BAY 41-2272 in streptozotocin-induced diabetic rats demonstrated that NO relaxation responses were reduced and the diminished relaxation responses were potentiated by BAY 41-2272, but not by sildenafil or NCX-911 [67]. These results provided further evidence for the effectiveness of sGC stimulators in ED that presents concomitantly with disease states involving impaired NO release.

In addition to heme-dependent stimulators, an NO and heme-independent class of sGC activators (BAY 58-2667, BAY 60-2770 and HMR 1766/S3448) has been introduced that target an altered form of sGC associated with many disease states, although low levels of heme-free sGC have been reported in normal physiologic states as well [68–70]. Initial characterization of the actions of the heme-independent sGC activator BAY 58-2667 demonstrated potent baseline stimulation, but only additive effects with NO donors on purified sGC activity [68, 71, 72]. Interestingly, it was shown that a significant increase in bioactivity occurs with oxidation of sGC by ODQ or removal of the heme complex from the enzyme [68, 71]. BAY 58-2667 has been reported to preferentially target the heme pocket of sGC and alter the enzyme to resemble the NO-active form [73]. Although it is accepted that oxidization weakens the binding of sGC to the heme prosthetic group, it remains controversial if the sGC activator removes the weakened prosthetic group before inserting itself or if the bioactivity is dependent on an enzyme that has already lost the heme complex [69, 74].

sGC activators have been shown to have beneficial effects in animal models of CVD, liver fibrosis, renal disease, ischemia-reperfusion-induced injury, thrombosis, systemic hypertension and pulmonary hypertension [73]. The use of sGC activators for ED has not been determined and pilot studies are needed to assess their utility in the treatment of this disease. The oxidative stress present in many disease states that present with ED suggests a potential role for the use of these activators in treating ED. The use of both sGC activators and stimulators is promising in conditions of altered heme conformation as well as in conditions where NO synthesis is impaired such as diabetes, hypertension, or prostatectomy in which there was neural damage [54, 59, 66, 75–78].

## 7. Rho-Kinase Inhibitors

When the penis is flaccid, cavernous smooth muscle as well as smooth muscle of arterioles is predominantly contracted and allows minimal arterial inflow to the tissues [79]. Smooth muscle contraction and relaxation is related to the level of free cytosolic calcium in the cell. Norepinephrine, endothelin-1 and prostaglandin F2*α* activate receptors on smooth muscle cells to increase intracellular levels of inositol triphosphate (IP_3_) and diacylglycerol (DAG) via a phospholipase C (PLC) mediated pathway. The accumulation of these intracellular messengers facilitates the release of Ca^2+^ from store and the opening of calcium channels on the cell membrane. The increase of intracellular Ca^2+^ concentration results in calcium binding to calmodulin and activation of myosin light chain kinase [80]. Phosphorylated myosin light chains trigger cycling of myosin crossbridges along actin filaments and generation of force as well as activation of myosin ATPase which hydrolyzes ATP to provide necessary energy for contraction.

When intracellular calcium levels return back to basal level, a sensitization pathway takes place with RhoA and Rho-kinase. RhoA is a small, monomeric G protein that activates Rho-kinase. Rho-kinase phosphorylates and inhibits the regulatory subunit of myosin phosphatase within smooth muscle cells. This action maintains phosphorylation of myosin filaments and contractile tone within the smooth muscle [81]. Vasodilation of arteries in the corpora is largely responsible for mediating the erectile process, and inhibition of the calcium sensitization pathway with Rho-kinase inhibitors offers a therapeutic option for the treatment of ED that does not involve the direct targeting of the NO/sGC/cGMP pathway.

Ic injections of the Rho-kinase inhibitor Y-27632 in rats pretreated with NOS inhibitors (L-NNA and L-NAME) or sGC inhibitors (methylene blue and ODQ) resulted in increased erectile activity in response to nerve stimulation seemingly independent of NO [82]. It was also proposed that NO may act to inhibit the RhoA/Rho-kinase pathway in the normal erectile response [83, 84]. Increases in intracavernosal pressure (ICP) were observed with ic injections alone without nerve stimulation, which provided evidence for a constant role for the RhoA/Rho-kinase pathway in maintaining flaccidity in the penis [82]. Rat cavernosum transfected with an adeno-associated viral gene dominant negative RhoA mutant (T19NRhoA) showed enhanced erectile activity providing further support for the role of RhoA in maintaining the flaccid state of the penis [85]. Western blot analysis in human corpus cavernosum tissue verified the presence and activity of RhoA/Rho-kinase in human penile cavernosal smooth muscle [86].

It has been shown that endothelial dysfunction and impaired NOS activity in the corpora cavernosum largely contributes to ED in diabetic men [87, 88]. A mechanism for diabetes-induced erectile dysfunction was demonstrated in which upregulated RhoA/Rho-kinase levels were found in cavernosal tissue of streptozotocin-induced diabetic rats. Erectile activity, cavernosal eNOS protein, constitutive NOS activity, and cGMP levels were restored to levels found in control animals after transfection with a dominant negative RhoA mutant [89]. Chronic administration of the Rho-kinase inhibitor fasudil was shown to prevent vasculogenic ED while reducing levels of pelvic atherosclerosis in a rat model receiving atherosclerosis-prone treatments [90]. A more recent study suggested that diabetic-associated ED due to upregulation of the penile RhoA/Rho-kinase pathway enhances PTEN/Akt activity leading to corporal apoptosis [91]. The study also suggested that chronic administration of the Rho-kinase inhibitor fasudil is more effective at reversing these detrimental biochemical changes than insulin administration in diabetic rats [91]. Further research is needed to determine if the reduced atherosclerosis and improved erectile activity could be observed in patients with severe CVD and/or diabetes.

Human clinical trials have recently been performed using SAR407899, a Rho-kinase inhibitor four times more potent than fasudil, for the treatment of mild to moderate ED (Rhoket trials—Aventis). Statins are used clinically as powerful lipid lowering agents. These compounds also have the ability to block the formation of isoprenoid intermediates required for RhoA activity [92]. It was recently shown that low-dose atorvastatin (Lipitor) normalizes the diabetic response to sildenafil in STZ-treated diabetic rats suggesting an inhibitory role of statins upon the RhoA/Rho-kinase pathway [93]. A clinical trial was recently concluded demonstrating the safety of the concurrent use of the PDE-5 inhibitor vardenafil with statins, thus offering a potential benefit to many men suffering from dyslipidemic conditions such as Type II diabetes and ED [94].

## 8. Intracavernosal Sodium Nitrite as a Novel NO Donor

It has been hypothesized that the nitrite anion is an important reservoir of circulating NO. Nitrite was used clinically by Brunton and co-workers for the treatment of angina pectoris and for lowering blood pressure a century and a half ago [95–97]. In animal models nitrite has been shown to mediate pulmonary vasodilation and prevent ischemia-reperfusion injury in the brain, liver, and heart [98–100]. The use of intracavernosal NO donors such as SNP to treat ED has been controversial because of hypotensive side effects [101, 102]. We have recently shown in the rat that sodium nitrite (NaNO_2_) administered ic increases ICP, decreases systemic arterial pressure and is 1000-fold less potent than the NO donor SNP [103]. Experiments with the NOS inhibitor L-NAME and the xanthine oxidoreductase (XOR) inhibitor allopurinol suggest a mechanism of nitrite bioactivation in the corpora that is mediated through eNOS, whereas nitrite bioactivation in the systemic vascular beds is largely due to the activity of XOR [100, 103]. The ability of nitrite to enhance erectile activity suggests further investigation in the use of nitrite as a therapeutic agent for ED.

## 9. Conclusion

The worldwide prevalence of ED in society requires new treatments for the growing number of patients with comorbid conditions that have become refractory to typical PDE-5 therapy. In the past ten years, the emergence of sGC stimulators/activators, Rho-kinase inhibitors and novel NO donors offers therapeutic potential for patients in which NO release and/or synthesis is impaired. Further research is needed to demonstrate the safety and efficacy of these therapies for the treatment of ED.
